# Perspectives and Experiences of Smartphone Overuse among University Students in Umm Al-Qura University (UQU), Saudi Arabia: A Qualitative Analysis

**DOI:** 10.3390/ijerph19074397

**Published:** 2022-04-06

**Authors:** Mohammad Saud Alotaibi, Mim Fox, Robyn Coman, Zubair Ahmed Ratan, Hassan Hosseinzadeh

**Affiliations:** 1 Department of Social Work, College of Social Sciences, Umm Al-Qura University, Mecca 24382, Saudi Arabia; msmasoud@uqu.edu.sa; 2 School of Health and Society, Faculty of the Arts, Social Sciences and Humanities, University of Wollongong, Northfields Ave., Wollongong, NSW 2522, Australia; mfox@uow.edu.au (M.F.); rcoman@uow.edu.au (R.C.); zar241@uowmail.edu.au (Z.A.R.); 3 Department of Biomedical Engineering, Khulna University of Engineering and Technology, Khulna 9203, Bangladesh

**Keywords:** smartphone overuse, university students, university staff, risk factors, impacts of smartphone overuse

## Abstract

Smartphone overuse and addiction is a growing concern worldwide. However, there are limited studies about smartphone addiction and its impacts on university students in Saudi Arabia. This qualitative study aimed to elicit students’ and university staff’s perspectives and experiences about smartphone overuse/addiction in Umm Al-Qura University (UQU), Saudi Arabia. Fifteen undergraduate students and 18 university staff (13 lecturers and five professionals) were recruited for the purpose of this study. The study data were collected using semi-structured interviews and analysed using thematic analysis. The qualitative data comprising 33 participants (students and staff) identified four major themes including the perception of smartphone use; causes of smartphone overuse; negative impacts of smartphone overuse; and strategies to reduce the overuse of smartphone. The overall findings confirmed that students and staff alike held both positive and negative perceptions about using a smartphone. Potential factors leading to smartphone overuse included personal factors (extended free time and low self-confidence, irresponsibility/escaping certain social gatherings/passing the time); smartphone factors (reasonable price, attractive advertisements (ads), and engaging smartphone Apps); and social factors (social pressure and fear of losing a connection). The main negative impacts of smartphone overuse were found to be related to low academic productivity, poor physical health (body pain, lack of sleep, and low exercise), compromised mental well-being (stress and negative emotions), and decreased socialisation (social isolation and a reduction in face-to-face communication). Our findings suggested that awareness campaigns about smartphone overuse, promoting family and social events, encouraging physical activities, and limiting internet use can reduce smartphone usage among university students. This finding has significant implications for decision-makers.

## 1. Introduction

Smartphone use has been exponentially increasing among university students worldwide [1], a recent global consumer survey found that 93% of adults aged 18 to 24 had the highest smartphone ownership [2]. Smartphones are being considered an integral part of daily life, importantly, smartphones afford students ample opportunities to obtain useful information anytime, anywhere [3]. Students use this device to access teaching content and collaborate with both teachers and peers [4]. However, it has been also observed that instead of attending lectures, students continue texting throughout and are unable to recall the lecture content [5]. In fact, smartphone overuse discourages face-to-face interactions [6], which can precipitate or exacerbate various physical and mental disorders [7,8]. Studies reported that smartphone ownership among students was particularly high in certain countries; most notably, Ghana (74%), Australia (81%), the UK (83%) [9], the US (91%) [10], and 94–99% in Saudi Arabia [11,12,13]. 

Due to the flexibility and mobility of smartphones, students can access the internet anywhere; this allows them to search for their study-related materials or obtain general information [14]. Smartphones provide a platform which facilitates learning through the use of specific applications which support university education [15]. Communication applications and social networking sites likewise foster the rapid dissemination of important information, while faster communication between students and faculty staff enhances effective study and productive collaboration [10]. In recent years Saudi Arabia has become an increasingly affluent and literate society [16]. Saudis are now among the top users of smartphones in the world. Many Saudis equate the use of smartphones with a newer contemporary lifestyle. As such, Saudis welcome the power of social media platforms as a means to express themselves, to share their lifestyle, and to communicate social issues [17]. Demographically, approximately 70% of the population of Saudi Arabia is under 34 years old, 5,802,334 of which are in the 20–35 age range which corresponds to the age range of Saudi university students. In Saudi Arabia, there are currently over 1.7 million students enrolled in universities and colleges [18]. Studies from two different Saudis universities concur that almost all university students had smartphones [11,12] and 95% of university students used smartphones to access social media platforms in 2016 [19]. 

Mobile learning is arguably the next phase of e-learning [20]. The level of awareness and adoption of modern technology is gradually increasing as mobile technology brings about a new mode of learning for people with a higher level of flexibility. Rapid development in the functionalities of mobile technology has stimulated the performance of a variety of tasks on a smartphone device. The reduction in the cost of smartphones has further encouraged using smartphones for education purposes [21]. In Saudi Arabia, the Ministry of Education has formally adopted e-learning. This encourages Saudis to study online through the use of certain smartphone applications; especially those students living in small cities or remote areas [22]. 

As smartphones have become ubiquitous, their use has increased significantly in recent years, particularly among the young [23]. Many young individuals have even been observed engrossed in their smartphones while walking in the street [24]. In fact, many are becoming nomophobic: “the psychological fear of being detached from mobile phone connectivity” [25]. Meanwhile, smartphones encourage constant use of social media, unlimited texting, video gaming, and online shopping, and so on; all of which may culminate in smartphone overuse [26]. Lee, et al. [27] assert that smartphones have become the last thing users look at before going to sleep, and the first thing they look at in the morning. Many users, particularly the young, have become so dependent on smartphone devices that they rely on them to establish new social connections and exchanging pictures [4]. “Boredom proneness” has also been posited as another prominent determinant of smartphone addiction [28] and a recent study suggested that university students may turn to their smartphones to relieve inertia or monotony [29]. In short, when university students feel bored or have little to occupy their time, they fall back on excessive smartphone use for diversion. Moreover, another study concluded that smartphones can be used as an entertainment tool that enables students ‘escape’ from uncomfortable situations [30]. Thus, in many cases, smartphones are a means to avoid daily problems, and to relieve feelings of depression, helplessness, and guilt [31]. In particular, students often use smartphones to construct their own reality: a form of existential denial which can create and/or intensify social isolation [26].

According to Ching, et al. [32], smartphone addiction is “mainly characterised by excessive or poorly controlled preoccupations, usage or behaviour regarding smartphone use; to the extent that individuals neglect other areas of life”. Addiction is not an easy concept to pin down. Moreover, since it is arguably pejorative, the application of the term “addiction” can be, in and of itself, highly controversial [33]. That being said, central to any definition is dependence on a substance or activity. According to Alhazmi, et al. [34], “the excessive use of smartphones to a level where it interferes with the daily lives of users is considered to be smartphone addiction”. Smartphone addiction creates common mental disorders, including suicide ideation [35], poor sleep patterns [36,37,38], and various depressive symptoms [39,40,41,42], all of which have serious implications for mental well-being. While the term “addiction” has been largely applied to substance addiction (e.g., alcohol, drugs), other behaviour patterns such as gambling, drug abuse, computer gaming, internet browsing, or even excessive chatting, can form habits which ultimately mutate into the obligatory behaviours of addiction [33]. Both substance and non-substance addiction have similar adverse consequences for behavioural patterns, emotional well-being, and healthful physiology [43]. Smartphone addiction refers to behaviour addiction and growing evidence demonstrates that behaviour addiction can precipitate mental disorders with the attendant social, cultural, and economic consequences [44,45]. For instance, excessive internet gaming may lead to adverse outcomes on psychological well-being, real-life social activity, offline social support, and academic performance [46]. In light of this, such behavioural addictions are now recognised as mental disorders [44,47]. Specifically, two types of behavioural addictions have been officially categorised by the Diagnostic and Statistical Manual of Mental Disorders, Fifth Edition (DSM-5) under non-substance related behavioural addiction: namely, gambling and internet gaming disorders [47]. 

The existing literature reported that smartphone overuse can lead to social isolation [48,49] or reduce face-to-face communication [50,51]. A recent study from Kuwait demonstrated a significant relationship between a high level of social isolation and smartphone addiction among university students [48]. The demographic surge in the proportion of young Saudis has brought about rapid development and growth in education and acceptance of technology in Saudi Arabia. As such, the use of smartphones as part of technological development has seen a significant expansion and increase in the Kingdom, particularly among students. There is a paucity of knowledge about smartphone overuse among the university students and this study aims to explore the ideas and perceptions of smartphone overuse among university students from Saudi culture which will help policymakers to take necessary interventions against smartphone addiction. 

## 2. Methods

### 2.1. Study Design and Data Collection

This was a qualitative study conducted among undergraduate students and university staff at Umm Al-Qura University (UQU) in Makkah, Westering Province, Saudi Arabia, between June 2019 and February 2020. Both students and staff were included to provide a comprehensive picture of smartphone use and its consequence in a university setting. A purposive sampling technique was used to collect qualitative data from the eligible participants. Purposive sampling allows the researcher to identify and select participants who meet the inclusion criteria for the purpose of a study [52]. Purposive sampling was also used to achieve maximum variation in key characteristics such as gender, education, and age. Qualitative data was collected from university students who were enrolled in an undergraduate degree at UQU, along with university staff comprised of lecturers and professionals responsible for student academic performance and well-being at UQU. Semi-structured interviews were used to collect data from male participants while an open-ended interview questionnaire was used to collect data from female participants. Due to religious and cultural restrictions in Saudi Arabia, female participants were unable to participate in interpersonal semi-structured interviews with the male researcher. As a result, an open-ended interview questionnaire (using the same question schedule as the semi-structured interviews) was designed for female participant use. Semi-structured interviews and interview questionnaires continued until data saturation was reached and no new findings emerged [53].

All eligible participants were provided with a Participant Information Sheet (PIS) and Participant Consent Form (PCF) outlining the study’s aims and processes, voluntary participation, withdrawal right, confidentiality, publication of the research results, and data storage details, and benefits of research. Informed consent was obtained from all respondents prior to participation in the study. Data collection and recruitment were conducted between June 2019 and February 2020.

The university students were asked for their views on the role of smartphones in their daily life; their thoughts on overusing a smartphone and its contributing factors; the signs of smartphone overuse; and their ideas/experiences on how to prevent smartphone overuse. 

The university staff was asked for their perspectives on smartphone addiction among students; whether they perceive smartphone addiction to be a problem among students; observed effects of smartphone addiction on their student’s performance; potential factors that could lead to smartphone addiction among students; smartphone addiction prevention; challenges students may face if they try to reduce their smartphone usage; and whether the university provides assistance for smartphone overuse/addiction among students. 

### 2.2. Participants

A total number of 33 eligible participants were interviewed. A total of 15 university students (8 male/7 female), aged 20–30 years old, mostly of single status (*n* = 12), and not gainfully employed (*n* = 14), participated in the qualitative data collection. A total of 18 university staff (5 male/13 female) participated in the interviews, aged 26–52 years old, most of them held PhD degrees (*n* = 12), and their teaching or professional experience ranged from 2–33 years. Thirteen were lecturers and five were professionals responsible for student academic performance and well-being.

### 2.3. Data Analysis

Qualitative data analysis comprised two main stages: data preparation; and data analysis. In the data preparation stage, all of the qualitative data was transcribed and translated from Arabic into English. The transcripts for each participant were saved and coded in a separate Word document file. In the data analysis stage, thematic analysis was used to identify themes drawn from student and university staff perspectives and experiences about smartphone use/addiction. An “inductive thematic analysis” was done for the data analysis [54], by completing six steps: (1) becoming familiar with data through repeated readings; (2) creating initial codes by coding interesting information and collating information relevant to each code; (3) searching for themes by collating all codes into initial themes or sub-themes; (4) reviewing themes by (re)checking the pattern of each theme or sub-themes in relation to the coded extracts in the entire data set in order to map and generate a thematic analysis; (5) ongoing analysis to define and refine the specifics of each theme and generate clear definitions and names for each theme; and (6) producing the report by writing up a narrative story by using selected quotations which capture the essence of the demonstrated themes [54]. 

Following a close reading of all of the participant transcripts, the data was categorised under a set of initial codes. A descriptive explanation of each code was provided to ensure each one was unique and relevant participant quotations were entered under each code. The codes were then assigned to appropriate sub-themes and themes. The coding process was confirmed several times before determining the final results to ensure the data was interpreted accurately and represented the participant perspectives. Since male participants were interviewed in person, their quotes were coded as “face-to-face interview” whilst female participants responded to an open-ended interview questionnaire their quotes were coded as “written response”.

## 3. Results

The findings of the thematic data analysis resulted in the identification of four main themes: perception of smartphone use; causes of smartphone overuse; negative impacts of smartphone overuse; and strategies to reduce overuse of smartphone. Under each theme, a further group of subthemes and codes was identified. 

### 3.1. Perception of Smartphone Use

**Figure 1:**
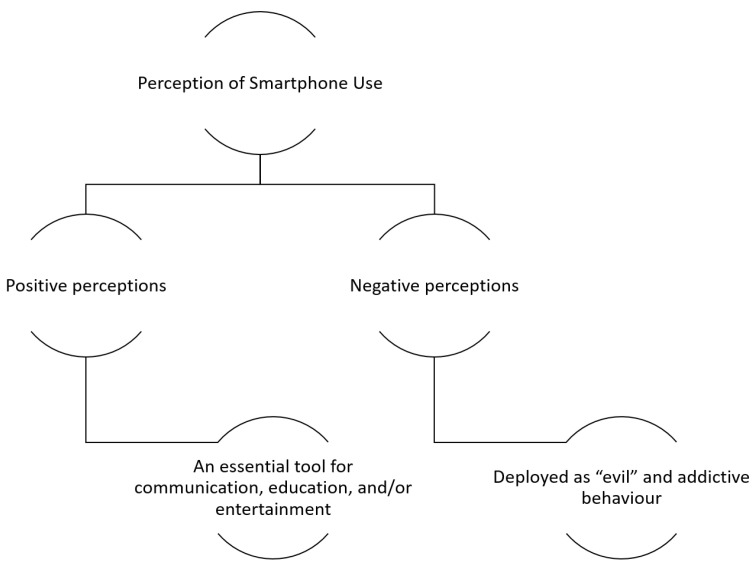
Perception of Smartphone Use.

Both university students and staff reported their perceptions and offered their opinions regarding smartphone usage. Two further sub-themes emerged within this overarching theme: namely, positive perceptions; and negative perceptions. Participants who held positive perceptions considered it an essential tool for communication, education, and/or entertainment. However, those who held negative attitudes towards smartphones considered it an adverse phenomenon and claimed that smartphone technology can be harmful and deployed as “evil” and addictive behaviour such as using the smartphone in class, in meetings, and upon waking in the morning. (see Figure 1).

#### 3.1.1. Positive Perceptions

The university student data indicated that the participants considered the smartphone an essential and useful tool. Most university students maintained that smartphones are an essential part of their daily life. They confirmed that smartphones have become ingrained in their generation and have become part of their normal lifestyle. For example, one university student said:

*The role of the smartphone in our generation… is different from the previous generation. This generation considers mobile* (smartphone) *to be essential.*

(Student N-1, face-face interview)

Another emphasised how important it was for him to check his smartphone upon waking every morning:

*I see it* (smartphone) *has become one of the most important things. I mean when anyone wakes up, they directly check their phone. It is the first and the most important thing for everyone now.*

(Student N-7, face-face interview)

However, university students defended smartphone use as beneficial for communication, education, and entertainment. They described how the smartphone is used in keeping in touch, exchanging cultures with others, or keeping them updated. For example, one student indicated that:

*There is no doubt that the smartphone has a prominent role in our daily lives by keeping us in touch with others, keeping us informed about the cultures around us, and informing us about the most important news.*

(Student N-9, written response)

Other university students provided examples of how they used the smartphone for education purposes in terms of reading books and doing homework; saving considerable time and effort. As one student explained:

*It* (smartphone) *has a big role in providing us with information, also facilitating access to books, references, and sources. It is saving time, effort, and money.*

(Student N-9, written response)

In addition, one student remarked that they use smartphones to entertain themselves after completing their daily tasks:

*Sometimes when I have free time after finishing home tasks and work appointments, I use my smartphone to entertain myself.*

(Student N-6, face-face interview)

University students defended smartphone use as beneficial for communication, education, and entertainment. They described how the smartphone is used in keeping in touch, exchanging cultures with others, or keeping them updated. For example, one student indicated that:

*There is no doubt that the smartphone has a prominent role in our daily lives by keeping us in touch with others, keeping us informed about the cultures around us, and informing us about the most important news.*

(Student N-9, written response)

The perspectives of the university staff echoed those of the university students. University staff maintained that the smartphone is now an essential tool for university students and the younger generation due to the many advantages embedded in its use. They concurred that smartphones enhance communication, provide access to entertainment, and a platform for sharing ideas and opinions. One staff member summarised why students use smartphones:

*A smartphone contains entertainment, gaming, and social communication. They* (students) *can contact anyone without any limitations. Students can contact social media figures, sports players, politicians, actors, or famous people. They* (students) *may also express their opinions.*

(Staff N-18, face-face interview)

The university staff generally focused on the advantages of smartphone use within the education system, and as a useful tool that enables students to extend their learning and knowledge. As one staff member pointed out:

*The positive side* (of using smartphones) *is that students can use this technology to obtain information and facilitate their learning.*

(Staff N-13, face-face interview)

Another staff member considered smartphone technology beneficial if correctly used by university students:

*If smartphones are properly used, they do not pose a problem. Students can use smartphones to communicate, access news, and entertain themselves all in one device.*

(Staff N-2, written response)

The university staff generally focused on the advantages of smartphone use within the education system, and as a useful tool that enables students to extend their learning and knowledge. As one staff member pointed out:

*The positive side* (of using smartphones) *is that students can use this technology to obtain information and facilitate their learning.*

(Staff N-13, face-face interview)

Another outlined how smartphones are now regularly used by younger students as a tool to take notes, record lectures, access information, and even complete homework assignments:

*I have noticed that students are using* (their) *phones for taking notes, and recording lectures. No one uses a pen and paper anymore to write. They* (students) *are using their phones to access textbooks, and even to do homework.*

(Staff N-17, face-face interview)

A staff colleague agreed that:

*They* (students) *use it* (smartphone) *for browsing international electronic libraries.*

(Staff N-5, written response)

#### 3.1.2. Negative Perceptions

The university students reported a range of negative views of smartphones, with four claiming they can be used for “evil” behaviours such as insulting others or watching inappropriate movies or pictures. They also admitted to using their smartphone to an excessive degree. Several participants claimed some students engage in illegal or forbidden activities which contravene acceptable local customs, traditions, or religious orthodoxy. For example, one university student indicated that:

*Smartphones may be used for evil ways like watching what our God would not be pleased with* (forbidden) *and obscene things to others.*

(Student N-14, written response)

Another university student heartily agreed:

*I see it* (smartphone) *as more evil than good, depending on how we use it. You see people on the phone 24 h a day. It steals your time, your effort, and your health. Because you are constantly on the phone, you are not free to do anything else!*

(Student N-2, face-face interview)

University students reported feeling so attached to their smartphones that it is difficult to be away from it. As one student asserted:

*I cannot live without my phone. I mean, I use it everywhere; indoors and outdoors.*

(Student N-6, face-face interview)

Moreover, university students acknowledged that such smartphone overuse is now normal and habitual. One university student said:

*When I wake up in the morning, I must check the phone. Checking my phone has become a habit for me now.*

(Student N-1, face-face interview)

Another student agreed it was second nature:

*I see that it* (using smartphone) *has become a habit. When I wake up, I check my phone for one or two hours. I see it has become a habit.*

(Student N-7, face-face interview)

University students also reported that smartphones are being overused in class, at social events, and even while driving. For instance, one of the students stated that:

*They* (excessive smartphone users) *use the Bluetooth headset and cover it by wearing the Shemagh* (traditional head cover worn by men) *to watch movies in class.*

(Student N-1, face-face interview)

Another university student expressed the anger he felt whenever he saw students absorbed in their smartphones during lecture time:

*I see many of them* (students overusing smartphones) *busy during the lectures with their smartphones. Today in the lecture, I saw one of them chatting via smartphone, during the lecture… Also, I saw some of the students are playing PUBG* (video game) *in the class!*

(Student N-3, face-face interview)

Some university students noticed that students using smartphones excessively at social events, even when it is not socially or culturally acceptable. For example, one student said:

*It is unacceptable to play with the phone during social events throughout the time and not to engage in pleasant conversations during these meetings; especially with parents.*

(Student N-9, written response)

Surprisingly, some of the students openly admitted they even used smartphones in potentially life-threatening circumstances. One of the university students explained that he and his friends used their smartphones while driving:

*We are overusing it* (smartphone). *For example, when I am heading to the university, I have a magnetic phone holder for the car that I installed to watch videos while I am driving. Not only me! I noticed more than four, five, six, of my friends are doing the same, and watching YouTube videos. This is a big problem…*

(Student N-3, face-face interview)

Similarly, university staff had observed the increased usage of smartphones among university students.

*Smartphone usage spread among the students in an intensive manner that might even become an addiction. Students spend most of their day using their smartphones.*

(Staff N-5, written response)

Indeed, university staff agreed that smartphone addiction requires urgent intervention. As one staff member elaborated:

*Smartphone addiction among students is a huge problem. It wastes their time. It needs urgent intervention.*

(Staff N-10, written response)

University staff also noticed certain behaviours that reflect the overuse of smartphones among university students. One staff member complained that:

*Some students hide their phones below the table during lectures and keep chatting on WhatsApp.*

(Staff N-1, written response)

Another university staff member explained:

*Students spend their time mostly browsing smartphone applications; sitting near a power source to charge their smartphones. Even inside the class, where the rules do not allow it, and the punishments do not stop them. So how can we not see that it is an addiction?*

(Staff N-4, written response)

Also, two of the university staff expressed concern that using smartphones frequently exposes university students to cyberbullying activities:

*I noticed cyberbullying is spreading among university students due to access to smartphones.*

(Staff N-6, written response)

*Smartphone usage might expose them* (university students) *to cyberbullying, racism, and other negative behaviours.*

(Staff N-18, face-face interview)

### 3.2. Causes of Smartphone Overuse

**Figure 2:**
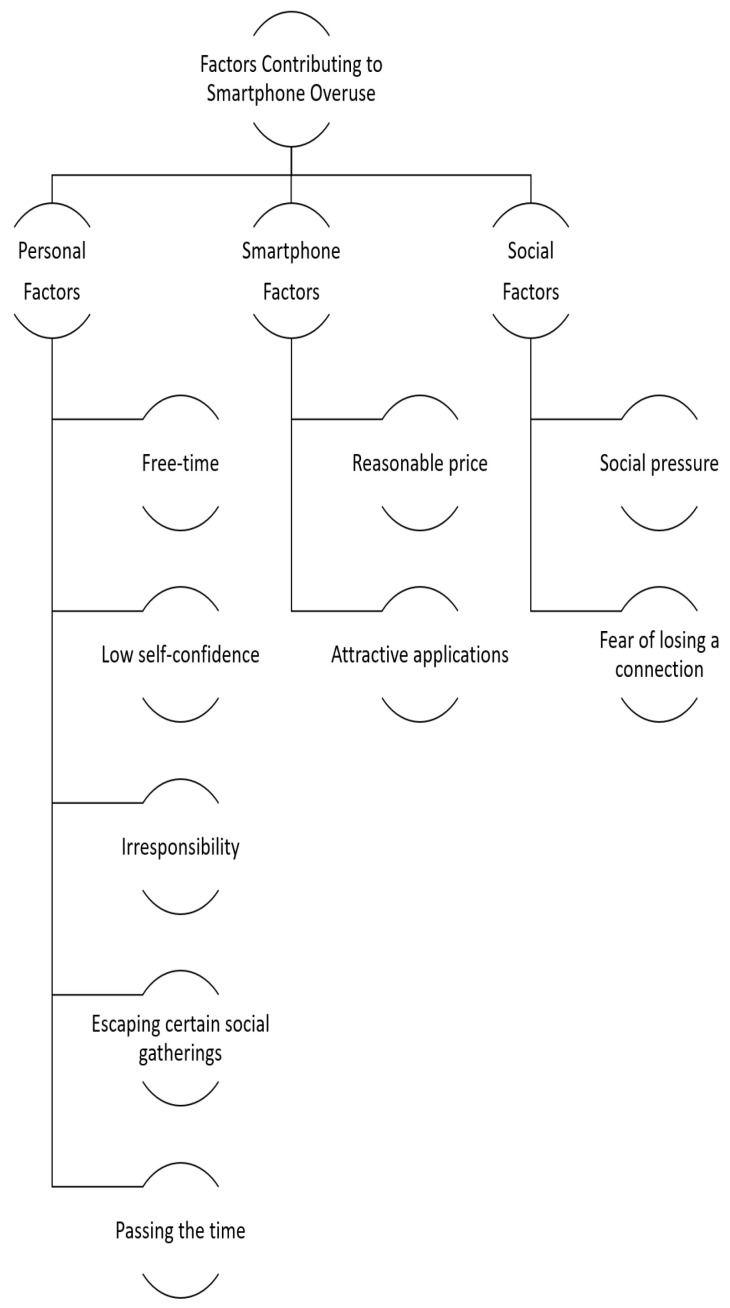
Causes of Smartphone Overuse.

The data indicated that three sub-themes or groups of factors which arguably contribute to the increasing use of the smartphone: namely, personal factors; smartphone factors; and social factors. Availability of extended free time, low self-confidence, and irresponsibility/escaping certain social gatherings/passing the time, were identified as the main personal factors. The availability of competitively priced smartphones and attractive applications of smartphones were also mentioned in relation to increases in the overuse of smartphones amongst students. Finally, a range of social factors was linked with the overuse of smartphones among students, including peer pressure, and fear of losing connection. These factors are delineated below from both student and staff perspectives. (see Figure 2).

#### 3.2.1. Personal Factors

The data indicated that extended free time, low self-confidence, lack of responsibility, and passing time during certain social gatherings, appeared to play a pivotal role in the overuse of smartphones. Most of the university students agreed that the availability of long periods of free time contributed to and encouraged the overuse of smartphones.

*In my opinion, free time is the biggest factor that contributes to our excessive use of smartphones.*

(Student N-9, written response)

Low self-confidence was also cited as a significant factor in overusing smartphones:

(Students) *who do not have the confidence to talk with people are more likely to use their smartphones all the time.*

(Student N-3, face-face interview)

Furthermore, students maintained that a lack of responsibility contributes to the overuse of smartphones among students:

*If someone* (student) *is careless and irresponsible. You will see them, a free guy without anything* (responsibilities). *They use a smartphone to keep themselves busy.*

(Student N-2, face-face interview)

Students also recognised that smartphones were sometimes used to avoid certain social interactions. For instance, one student admitted that:

*If I meet people I do not like to communicate with, I prefer to use my smartphone.*

(Student N-3, face-face interview)

The findings from the university staff found they largely agreed with university students in respect of most personal factors which can lead to the overuse of smartphones, including extended periods of free time, low self-confidence, and lack of responsibility. However, several staff drew attention to another significant factor: the lack of student knowledge regarding the impacts of smartphone addiction.

*Prolonged free-time, where the person* (university students) *cannot find useful stuff to do… they use their smartphone.*

(Staff N-12, written response)

University staff also linked the availability of prolonged free time with limited student participation in the usual social or entertainment activities. One staff member stated that:

*There are multiple factors that lead to smartphone addiction, such as an abundance of free- time for students and having no place to hang out.*

(Staff N-6, written response)

University staff maintained that low self-confidence could force students to choose online rather than interpersonal communication, which, in turn, could lead to smartphone overuse:

(Students) *feel comfortable presenting their ideas* (via smartphone-online) *without direct contact or disagreement*

(Staff N-3, written response)

Moreover, university staff pointed out that the university students’ frequent struggles to identify their life goals may also contribute to the overuse of smartphones:

*The absence of clear goals to pursue and work hard encourages students to use smartphones.*

(Staff N-2, written response)

Staff also speculated that lack of information about the negative consequences of smartphone addiction or overuse could play a significant role in smartphone addiction among students:

(University student) *does not understand the hazards of using smartphones for a prolonged time.*

(Staff N-3, written response)

*Lack of awareness of the impacts of smartphone addiction leads to the problem of excessive use of smartphones.*

(Staff N-9, written response)

#### 3.2.2. Smartphone Factors

The analysis confirmed several factors related to smartphones that can lead to overuse. These include the availability of affordable smartphone devices and interactive applications in the smartphones:

*Smartphones are cheap and easy to use.*

(Student N-13, written response)

(Smartphones) *are* (available) *at reasonable prices that enable everyone to have them.*

(Student N-15, written response)

*I see those games and applications: they are very attractive.*

(Student N-7, face-face interview)

University students also explained that the use of social media applications among Saudi university students could exacerbate smartphones overuse:

*Social media applications are the biggest reason makes us overuse our smartphones.*

(Student N-1, face-face interview)

Moreover, one student explained that smartphone games are particularly addictive for students:

*There are some games that make you so addicted. So that even if the internet is cut off, you must find a friend who connects you to the Internet.*

(Student N-4, face-face interview)

Similarly, university staff noted that the availability of smartphones and interactive applications results in the overuse of smartphones among students:

*Smartphones are easy to use; attractive and tempting, to the youth.* (University students) *are now tempted by many advertisements which attract them more and more to use their smartphones.*

(Staff N-18, face-face interview)

*When I spoke with students about the games on the smartphone, they told me that they use the games on a daily basis and for long hours.*

(Staff N-2, written response)

#### 3.2.3. Social Factors

Students unanimously cited social pressure as one of the social factors responsible for the drastic increase in the usage of smartphones:

*When a new movie is released, all of my friends start watching it* (via smartphone) *except me. I started thinking, “no, let me watch it with them”, so as not to be alone and feel old-fashioned.*

(Student N-1, face-face interview)

*Buying a new smartphone and using it everywhere has become fashionable.*

(Student N-5, face-face interview)

The results from university staff regarding social factors slightly diverged from that of students. While university staff largely agreed that social pressure is a huge factor in the overuse of smartphones, they added the additional factor of fear of losing a sense of connection:

*Mimicking others is one of the factors that lead to addiction. Students copy each other’s behaviour, and* (there is) *an urge to be admired through ‘likes’ and comments.*

(Staff N-6, written response)

*Fear of being called outdated or traditional forces them* (students) *to use a smartphone.*

(Staff N-1, written response)

University staff agreed that students keep using smartphones in order to maintain a constant connection with their friends:

*Fear of losing online friends pushes students to use their smartphone.*

(Staff N-1, written response)

### 3.3. Negative Impacts of Smartphone Overuse

**Figure 3:**
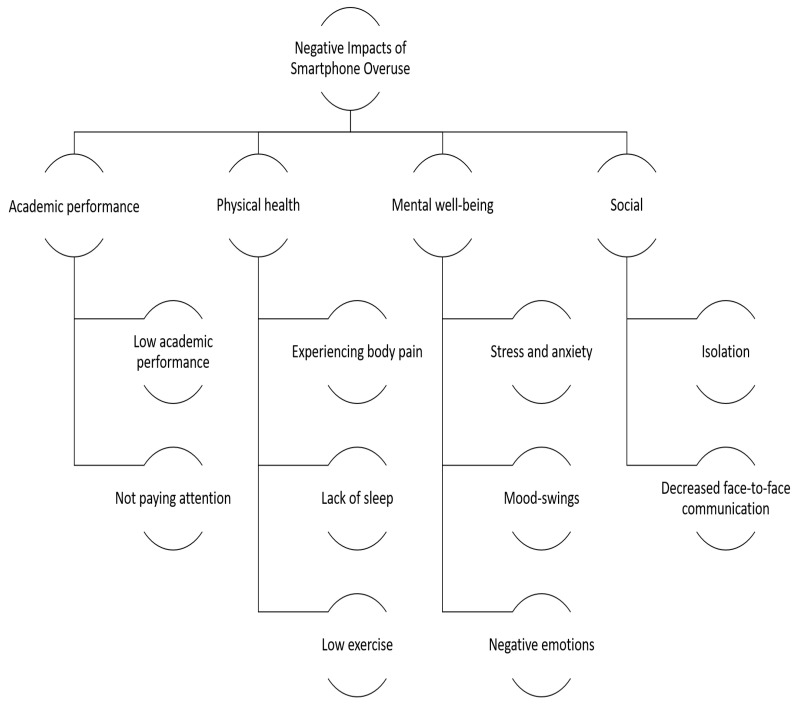
Negative Impacts of Smartphone Overuse.

The data collected identified four major negative impacts of the overuse of smartphones on students: namely, academic engagement and performance (low academic performance/not paying attention); physical (experiencing body pain, lack of sleep, and low exercise); mental well-being (stress and anxiety, mood swings and negative emotions); and social (isolation and decreased face-to-face communication). (see Figure 3).

#### 3.3.1. Academic Engagement and Performance

Students indicated that the overuse of smartphones can cause low academic productivity or performance and distraction from attention during both lectures and private study:

*Student academic performance is affected by smartphone use during lectures. It* (smartphone) *disrupts students’ focus in lectures.*

(Student N-5, face-face interview)

In addition to the impact on academic performance, university students admitted that using a smartphone distracts them from their daily tasks or jobs.

(Students) *who are excessive* (smartphone) *users, may forget their prayers, works, their important task, and other duties in their lives.*

(Student N-2, face-face interview)

Data analysis indicated that university staff focused on the negative impacts of the overuse of smartphones on student productivity; particularly student academic performance. The majority of staff members were concerned that smartphones usage persistently diverts student attention from their studies:

*It contributes to the distraction of students’ attention…*

(Staff N-7, written response)

*It* (a smartphone) *negatively affects the quality of students’ homework, as it consumes most of the students’ time and distracts their focus during homework.*

(Staff N-3, written response)

#### 3.3.2. Physical Factors

Students indicated that the overuse of smartphones exerts negative impacts on physical health resulting in body pains, lack of sleep, and inadequate exercise:

*There are some health concerns about the excessive use of smartphones including problems with vision, spasticity of the hand, and neck pain.*

(Student N-13, written response)

*I do not sleep well because notifications* (from smartphone applications) *keep waking me up.*

(Student N-1, face-face interview)

*My lack of exercise is because all of my time is occupied by using my smartphone.*

(Student N-4, face-face interview)

University staff participant responses echoed those of students. The data indicated that overuse of smartphones negatively impacts student’s physical health in terms of body pain, lack of sleep, and inadequate exercise:

*Using it* (smartphone) *for a prolonged time will affect eyesight and lead to neck pain, and low exercise.*

(Staff N-16, face-face interview)

*Mostly it* (smartphone) *negatively impacts sleep and physical activity levels.*

(Staff N-15, face-face interview)

#### 3.3.3. Mental Well-Being Factors

Students maintained that the overuse of smartphones may be associated with a range of negative emotions including stress, nervousness, tension, and anger. Several observed elevated signs of stress and nervousness amongst students who overuse a smartphone:

(Smartphones) *cause psychological problems such as nervousness.*

(Student N-8, face-face interview)

*Some smartphone games make us angry.*

(Student N-9, written response)

University staff held similar views to the student participants and agreed that the overuse of smartphones can result in stress, anxiety, and mood swings:

*Students often feel anxious and stressed out if the phone* (smartphone) *is taken away from them or the internet servers are disconnected.*

(Staff N-12, written response)

#### 3.3.4. Social Factors

Students indicated that the overuse of smartphones may negatively impact user’s social interaction, thereby fostering social isolation and reduced interpersonal communication:

*Smartphones affect students’ social life and isolate them from society.*

(Student N-11, written response)

We *received many criticisms about being isolated and not interacting with others because of using our smartphones all the time.*

(Student N-15, written response)

*Everyone around you is on their mobile: there is no conversation between them.*

(Student N-4, face-face interview)

The university staff’s responses resonated with those of students. Most of the university staff maintained that the overuse of smartphones may lead to social isolation:

*It* (smartphone) *also might lead to social problems causing isolation and not being integrated into society.*

(Staff N-6, written response)

*It* (smartphone) *disconnects the student from real life and social connections.*

(Staff N-8, written response)

### 3.4. Strategies to Reduce Smartphone Usage

**Figure 4:**
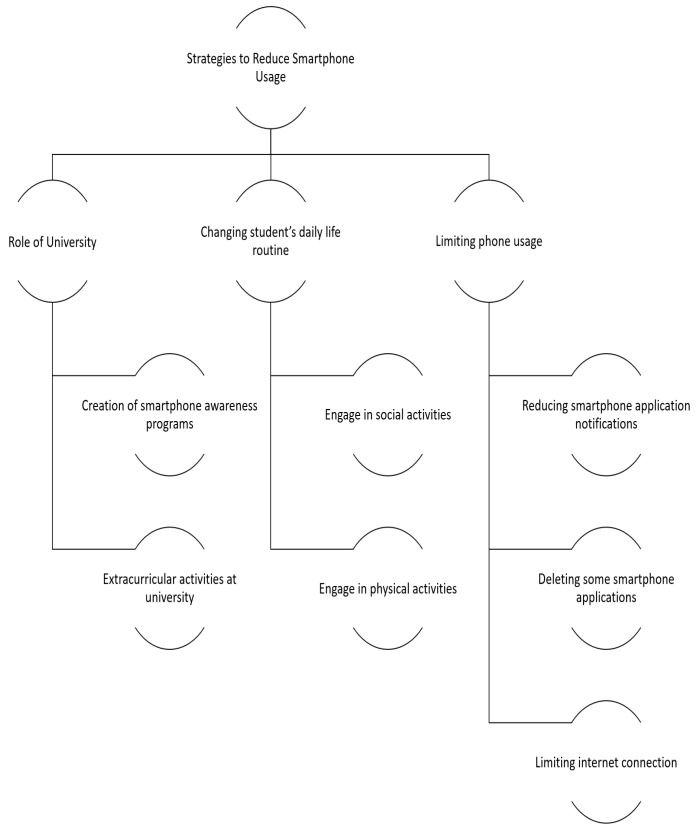
Strategies to Reduce Smartphone Usage.

The participants suggested various strategies for reducing the overuse of smartphones among university students. Some of those proposed included the creation of awareness programs, extracurricular activities, changing students’ daily life routines, and limiting phone usage. These suggestions are discussed below (Figure 4): 

#### 3.4.1. Creation of Smartphone Awareness Programs and Extracurricular Activities at University

Students suggested that the university could provide awareness programs and seminars to reduce the use of the smartphone by:

*… creating awareness programs at the university level such as programs, seminars, or sending text messages about smartphone overuse and addiction can improve students’ knowledge, and eventually decrease smartphone overuse and addiction among them.*

(Student N-1, face-face interview)

University staff also maintained that awareness programs, as well as extracurricular activities, could be used to educate university students about smartphone overuse and addiction:

*When students finish their lectures, they sit in the corridors and do nothing… So the university could provide extracurricular activities like drawing, writing, sports programs… Such programs can reduce phone use and prevent smartphone overuse or addiction.*

(Staff N-15, face-face interview)

*The university can help through awareness campaigns about smartphone addiction.*

(Staff N-9, written response)

*Providing field trips and entertainment journeys are great extracurricular activities to reduce smartphones among students.*

(Staff N-2, written response)

#### 3.4.2. Changing Students’ Daily Life Routine

The majority of students suggested that changing student’s daily routine to reduce free time could decrease smartphone overuse or addiction. They further held that engagement in physical activity, reading, and social activities can limit undue exposure to smartphones:

*Two weeks after going to the gym, I started sleeping enough. I wake up in the morning and sleep at night, with less smartphone use. My phone usage was reduced to five hours a day from nine hours or ten hours per day.*

(Student N-3, face-face interview)

*Socializing with people, like attending social events, can reduce phone usage.*

(Student N-7, face-face interview)

The majority of university staff agreed that changing daily life routines could prevent the overuse of smartphones among students:

*Encouraging students to participate in sports instead of spending their time on smartphones and social media can decrease smartphone overuse…*

(Staff N-1, written response)

Moreover, staff also suggested that social activities such as gatherings with family members or friends can help to reduce the overuse of smartphones among students:

*Participating in family and friends gatherings can help to decrease smartphone overuse.*

(Staff N-2, written response)

#### 3.4.3. Limiting Smartphone Usage

Students suggested that reducing smartphone application notifications, deleting some smartphone applications, and/or limiting internet connection could reduce smartphone overuse or addiction:

*Reducing smartphone applications’ notifications or deleting some applications or limiting smartphone use, or even disconnecting the internet can be used to reduce smartphone use.*

(Student N-9 written response)

*Installing some apps to count daily phone usage will help in controlling smartphone use…*

(Student N-13, written response)

University staff suggested that the overuse of smartphones could be reduced by limiting the usage of smartphones or preventing smartphones from being used in class:

*Defining a particular daily time period for not using any smartphone can help…*

(Staff N-12, written response)

*We could also limit internet access during lectures…*

(Staff N-18, face-face interview)

## 4. Discussion

Qualitative methods were applied in order to derive the broadest possible understanding of student experiences of using a smartphone and university staff perceptions of smartphone use among students. The overall findings confirmed that students and staff alike held both positive and negative perceptions about using a smartphone. Potential factors leading to smartphone overuse included personal factors, smartphone, and social factors. Finally, the participants suggested a range of strategies to reduce smartphone usage.

### 4.1. Perception of Smartphone Use

The perceptions of using a smartphone among university students and staff were found to be both positive and negative. Students and staff both maintain that smartphones are important in students’ daily lives especially in relation to *communication* (Staff N-18), *education* (Staff N-13), and *entertainment* (Staff N-18). This aligns with the literature which confirms university students hold predominantly positive attitudes towards smartphones and use them for education (e.g., reading lecture notes), communication, socialization, shopping, and internet searching [55,56,57]. 

Both students and staff cited the importance of smartphones in university education and agreed that smartphone technology can be harnessed for teaching and learning including recording lectures, accessing educational materials, sharing ideas, and online learning. Such positive attitudes towards using smartphones for education purposes among both students and staff confirm the enormous potential of smartphone technology to facilitate online tertiary education [56]. 

Nonetheless, students and staff expressed concern about smartphone overuse and addiction and the attendant sociocultural and health implications. They emphasised that the use of smartphones during social events or even in the presence of older people including parents or older siblings or relatives is considered socially unacceptable. Consistent with the literature, participants associated smartphone overuse with low levels of life satisfaction and quality of life [58]. 

Both students and staff purported that using a smartphone more frequently can lead to smartphone addiction. As one participant stated, *I see that it* (using smartphone) *has becomes a habit* (Student N-7). In line with this finding, a study modelling habitual and addictive smartphone behaviour of 386 respondents concluded that habitual behaviour use was positively correlated to smartphone addiction [59]. Student participants also mentioned that repetitively checking their phones on waking up in the morning, during lectures, and at social events, I cannot live without my phone. I mean, I use it everywhere; indoors and outside (Student N-6). These addictive smartphone patterns might be explained by the FoMO phenomenon, characterised by a constant deluge of updates and the desire to stay connected with others all the time [60]. 

Students reported that they were using smartphones while driving, *when I am heading to the university, I have a magnetic phone holder for the car that I installed to watch video while I am driving* (Student N-3), whereas staff was concerned about cyberbullying activities among smartphone users, *I noticed cyberbullying is spreading among university students due to access to smartphones* (Staff N-6). Previous studies associated traffic accidents involving pedestrians with using smartphones while driving [61,62]. For instance, a study in the United States showed that the number of pedestrian injuries increased from 559 to 1506 between 2004 and 2010 due to the increased use of smartphones in public places [63]. In line with our findings, a review study concluded that usage of smartphones is also more likely to be implicated in cyberbullying behaviours, whether as a target or a perpetrator [64]. 

### 4.2. Factors Contributing to Smartphone Overuse

Personal factors (extended free time and low self-confidence), smartphone factors (reasonable price, attractive ads, and engaging smartphone applications), and social factors (social pressure and fear of losing a connection) were identified as the main contributors to smartphone overuse among the participants.

In terms of personal factors, both students and staff mentioned that having longer periods of free time was a major contributing factor for increased smartphone usage among students. For instance, a student asserted that *free time is the biggest factor in my opinion that contributes to our excessive use of smartphones* (Student N-9), while a staff member commented that, … *the abundance of free time for students and having no place to hang out* (Staff N-6) encourages students to overuse smartphones. These findings are consistent with the literature which demonstrates that university students are more likely to overuse their smartphones when they have time on their hands [28,29,65,66,67]. Furthermore, and in line with the literature [68], low self-confidence appeared to be contributing to overusing smartphones among the participants, [students] who do not have the confidence to talk with people are more likely to use their smartphones all the time (Student N-3). 

In relation to smartphone factors, reasonable price, [smartphones] *are* [available] *at reasonable prices that enable everyone to have them* (Student N-15), attractive ads, and engaging Apps such as social media and gaming (*I see those games and applications: they are very attractive* (Student N-7), *social media applications are the biggest reason makes us overuse our smartphones* (Student N-1)) contributed to smartphone overuse. These findings are supported by literature indicating that social media and gaming Apps comprise the underlying factors of smartphone addiction among young users [26,69,70,71]. 

With regard to social factors, students indicated that peer pressure was the leading cause of smartphone overuse (*using it everywhere has become fashionable* (Student N-5), *Fear of being called outdated* (Staff N-1), and *mimicking others* (Staff N-6)) lead to smartphone overuse. However, staff participants cited the fear of losing online friends as a leading cause of overusing smartphones among students. These findings correspond with the findings of previous studies which concluded that social pressure [72,73,74] and fear of losing a connection [75,76] were triggers for smartphone addiction. For instance, a study conducted among 2820 Spanish smartphone users found that higher levels of social pressure were associated with higher levels of smartphone addiction [73]. 

### 4.3. Negative Impacts of Smartphone Overuse

The participants highlighted the negative impacts of smartphone overuse on students in four discrete areas: namely, academic performance, physical health, mental well-being, and socialization. 

Both students and staff concurred that overusing smartphones can exert negative effects on students’ academic performance. *It* (a smartphone) *negatively affects the quality of students’ homework, as it consumes most of the students’ time and distracts their focus during homework* (Staff N-3). Similarly, one student added that, (students) *who are excessive* (smartphone) *users, may forget their prayers, works, their important task, and other duties in their lives* (Student N-2). Consistent with the literature, overusing a smartphone culminates in poor academic performance [77,78]. 

Participants highlighted the negative impacts of overusing smartphones on the physical health of students including body pains (*there are some health concerns about the excessive use of smartphones including problems with vision, spasticity of the hand, and neck pain* (Student N-13)), lack of sleep (*I do not sleep well because notifications* [from smartphone applications] *keep waking me up* (Student N-1)), and physically inactive (*my lack of exercise is because all of my time occupied by using my smartphone* (Student N-4)). These findings are supported by a number of previous studies which demonstrated that overusing smartphones caused body pain [79,80,81], lack of sleep [36,37,38,82], and physical inactivity [83]. 

Mental health *symptoms (students often feel anxious and stressed out if the phone* (smartphone) *is taken away from them or the internet servers are disconnected* (Staff N-12)) and negative emotions (*some smartphone games make us angry* (Student N-9)) were also associated with smartphone overuse by the participants. Similarly, the findings from the literature review showed that stress, anxiety [41,84,85], and negative emotions [86,87] were related to smartphone addiction. 

The participants also perceived that the overuse of smartphones leads to diminished face-to-face interaction (*everyone around you is on the mobile: there is no conversation between them* (Student N-4)) and social isolation (*It* (smartphone) *also might lead to social problems causing isolation and not being integrated into society* (Staff N-6)). The finding of other studies confirms the negative effects of overusing smartphones on social isolation [48,49] and reductions in face-to-face communication [50,51]. 

### 4.4. Strategies to Reduce the Overuse of Smartphones

The participants believed that educational programs raising awareness about smartphone use, and promoting engagement in social, family, and physical activities, could reduce smartphone overuse among students.

The participants suggested that universities could reduce smartphone overuse in students by developing awareness programs *the university can help through awareness campaigns about smartphone addiction* (Staff N-9), offering social activities *providing field trips and entertainment journeys are great extracurricular activities to reduce smartphones among students* (Staff N-2), promoting physical activity *encouraging students to participate in sports instead of spending their time on smartphones and social media can decrease smartphone overuse* (Staff N-1), and encouraging family and friend activities *participating in family and friends gatherings can help to decrease smartphone overuse* (Staff N-2). 

Participants also suggested that reducing smartphone application notifications, deleting certain smartphone applications, and limiting internet connection could act to reduce smartphone overuse or addiction among students. *Installing some apps to count daily phone usage…* (Student N-13) and *limiting internet access during lectures* (Staff N-18) were both recommended as effective in preventing smartphone use during lectures.

Despite generating invaluable data regarding smartphone addiction and its main drivers, this study has some inevitable limitations. It was not possible for the male researcher to interview the female participants and record their interviews due to the social and cultural mores of the study setting. Thus, the written responses to open-end questions used with female participants may have constrained a more fulsome exploration of the perceptions of both student and staff female participants.

## 5. Conclusions

Students and staff held both positive and negative perceptions about using a smartphone. Personal factors (having free time and low self-confidence); smartphone factors (reasonable price, attractive advertisements (ads), and engaging smartphone Apps); and social factors (social pressure and fear of losing a connection) appeared the main potential factors leading to smartphone overuse among the participants. The main negative impacts of a smartphone overuse were found to be related to low academic productivity, poor physical health (body pain, lack of sleep, and low exercise), compromised mental well-being (stress and negative emotions), and decreased socialisation (social isolation and a reduction in face-to-face communication). The participants suggested that awareness campaigns about smartphone overuse, promoting family and social events, encouraging physical activities, and limiting internet use can reduce smartphone usage among university students. Findings for the qualitative data revealed how university students and staff perceive smartphone use in the Saudi Arabian context. Their real-world understanding of the leading factors of smartphone overuse and addiction among university students is of significant interest and value for both understanding smartphone addiction and devising specific real-world prevention interventions. The implications of the study are given below.

1. Develop policies and guidelines limiting the usage of smartphones during lectures.
2. Establish free and accessible sports facilities in all universities.
3. Develop specific counselling and prevention programmes with regard to smartphone overuse and addiction in universities.
4. Educate students about the proper use of social media.

## Data Availability

The data presented in this study are available on request from the corresponding author. The data are not publicly available due to privacy restrictions.
